# Thyroid functional parameters and correlative autoantibodies as prognostic factors for differentiated thyroid cancers

**DOI:** 10.18632/oncotarget.10236

**Published:** 2016-06-23

**Authors:** Chao Li, Wenbin Yu, Jinchuan Fan, Guojun Li, Xiaofeng Tao, Yun Feng, Ronghao Sun

**Affiliations:** ^1^ Department of Head and Neck Surgery, Sichuan Cancer Hospital, Chengdu, China; ^2^ Department of Head and Neck surgery, Key Laboratory of Carcinogenesis and Translational Research (Ministry of Education/Beijing), Peking University Cancer Hospital & Institute, Beijing, China; ^3^ Department of Head and Neck Surgery, The University of Texas MD Anderson Cancer Center, Houston, TX, USA; ^4^ Department of Epidemiology, The University of Texas MD Anderson Cancer Center, Houston, TX, USA; ^5^ Department of Radiology, Shanghai Ninth People's Hospital, Shanghai Jiao Tong University School of Medicine, Shanghai, China; ^6^ Department of Otorhinolaryngology Head & Neck Surgery, China-Japan Friendship Hospital, Beijing, China

**Keywords:** differentiated thyroid carcinoma, thyroid functional parameters, thyroid correlative autoantibodies, prognostic factors

## Abstract

To evaluate the effect of preoperative thyroid functional parameters and thyroid autoantibodies on aggressive clinicopathologic features and lymph node metastasis (LNM) of differentiated thyroid cancer patients. Four hundred twenty consecutive patients with initial surgery were enrolled from July 2010 to July 2015. The associations between aggressive clinicopathologic and LNM factors and thyroid functional & autoantibodies parameters were analyzed. Higher levels of TSH, TGAb or TMAb were found in patients with tumor size≥1 cm (all *P*<0.05), especially when TSH≥2.5 ulU/ml (*P*=0.03) and TGAb≥1 (*P*=0.01). Higher levels of TSH and TGAb and lower levels of T3 and T4 were found in patients with capsular invasion (all *P*<0.05), particularly when TSH≥2.5ulU/ml (*P*=0.03) and TGAb≥1 (*P*=0.005). The patients with multifocality had higher TAbs level (TAbs>1). Higher level of TSH was also found in patients with central LNM (*P*=0.001) and lateral LNM (*P*=0.002), especially with TSH≥2.5ulU/ml (*P*=0.003 and *P*=0.03). TGAb level was also found higher in patients with central LNM (*P*=0.02) and lateral LNM (*P*=0.01), especially with TGAb≥1 (*P*<0.05 and *P*=0.01). Higher level of TMAb was found in patients with lateral LNM (P<0.05). Moreover, multivariable analysis revealed that only TGAb was an independently predictive factor for primary tumor size≥1cm (*P*=0.01); and TSH level (*P*=0.01) and TGAb≥1 (*P*<0.05) were associated independently with central LNM. Thus, TSH level and TGAb≥1 were significantly independent predictors for central LNM, and might help make the decision of central neck dissection.

## INTRODUCTION

Differentiated thyroid cancer (DTC) is the most common malignancy in head and neck and endocrine system. The incidence of DTC continues to increase rapidly. DTC is usually considered to be an indolent tumor with low mortality and good prognosis. While a significant percentage of patients have persistent or recurrent disease, better prognosis mainly depends on appropriate treatments which rely not only on TNM staging but also on a risk stratification system.

Accurate risk stratification is needed to determine which patient would benefit from aggressive therapies. Clinicopathologic characteristics, including gender, age, radiation history, distant metastasis, bilateral nodularity, extrathyroidal extension, tumor size, tumor location, aggressive variants, are considered to be risk factors in the risk stratification system of DTC [1]. Some studies [2, 3] have suggested that these clinical factors were correlated with recurrence and metastasis of DTC, which directly affected patient's prognosis. The risk stratification system has been constantly improved recently, which has included more clinicopathologic characteristics, including molecular factors. More evidences [4, 5] suggested that a more aggressive clinical course could be expected in tumors which carried oncogenic mutations, such as BRAF V600E, TERT, or TP53 mutations. These somatic mutations have been included in the recently revised ATA guideline [6]. However, there have been fewer studies evaluating the associations between serum-based parameters and prognosis of DTC.

Serological parameters for DTC include thyroid functional parameters and correlative autoantibodies. Recently, it has been reported that the risk of thyroid malignancy was associated with some thyroid functional parameters, such as serum thyroid stimulating hormone (TSH) level [7]. However, there have been conflicting results regarding the roles of these thyroid functional parameters [8]. Serum thyroid autoantibodies (TAb), including thyroglobulin antibody (TgAb) and thyroid microsomal antibody (TMAb), are common in autoimmune thyroid disease (AIT). Some studies suggested that the presence of TAb increased 2-fold risk of DTC in patients with presence of TAb than in the general population [9]. However, this marker still has two drawbacks in clinical application. Firstly, the specificity of TAb is not satisfied. Secondly, the association between thyroid autoimmunity and prognosis of DTC remains controversial [10].

To assess the prognostic significance of preoperative thyroid functional parameters and thyroid autoantibodies on the treatment of DTC, in the present study, we analyzed prospectively collected data on preoperative TSH, thyroid hormones (T3 and T4), serum thyroglobulin (Tg) and correlative autoantibodies (TgAb and TMAb) among DTC patients before treatment. We evaluated the associations between these preoperative serological characteristics and postoperative aggressive clinicopathologic characteristics which might predict the prognosis of DTC. Additionally, the potential associations of these preoperative parameters with cervical lymph node metastasis (LNM) in DTC were assessed in this study.

## RESULTS

### Patient characteristics

The cohort of 420 patients consisted of 312 (74.3%) females and 108 (25.7%) males. The mean age of the patients was 42.8±13.5 (range 7-76) years. Among 420 patients, 93.6% (393/420) patients had papillary cancer, and 6.4% (27/420) had follicular tumors. The mean tumor size was 1.88 ± 1.34 cm. Of the 420 cases, pathological capsular invasion, multifocality, and bilateral affected lobes were found in 46.7% (196 of 420), 47.9% (201 of 420) and 32.6% (137 of 420) patients respectively. LNM were found in 276 (65.7%) patients, 247 (58.8%) patients in central compartment, and 185 (44.1%) in lateral neck. Twenty nine (6.9%) cases were confirmed to have lateral LNM, but not central nodes. The details of demographics, clinicopathologic characteristics, and mean levels of preoperative thyroid function, as well as autoantibodies of study patients were summarized in Table 1.

**Table 1:** Demographic and Clinicopathologic Characteristics of 420 Patients with DTC

Variables	No. of patients	%	Variables	No. of patients	%
Gender			Affected lobes		
Male	108	25.7	Unilateral	283	67.4
Female	312	74.3	Bilateral	137	32.6
Age(mean±SD,y)	42.8±13.5		Central LNM		
≥15 and <45	238	56.7	Yes	247	58.8
<15 or ≥45	182	43.3	No	173	41.2
Pathology			Lateral LNM		
Papillary	393	93.6	Yes	185	44.1
Follicular	27	6.4	No	235	55.9
Primary tumor size (mean±SD, cm)	1.9±1.3				
<1	123	29.3	T3(mean±SD, ng/ml)	1.6±0.8	
≥1	297	70.7			
Tumor location			T4(mean±SD, ng/ml)	84.1±22.9	
Upper pole	164	39.0			
Middle	134	31.9	TSH(mean±SD, uIU/ml)	2.1±1.5	
Lower pole	122	29.1			
Multifocality			Tg(mean±SD, ng/ml)	21.3±24.6	
Yes	201	47.9			
No	219	52.1	TGAb(mean±SD, ng/ml)	1.6±1.5	
Capsular invasion					
Yes	196	46.7	TMAb(mean±SD, ng/ml)	1.3±1.2	
No	224	53.3			

### Comparison of preoperative thyroid function and autoantibody levels according to tumor aggressive clinicopathologic features

We evaluated the tumor aggressive clinicopathologic prognostic features including primary tumor size, affected Lobes, capsular invasion and multifocality and the thyroid function parameters (T3, T4, TSH, and Tg). As shown in Table 2 and Table 3, the TSH level was significantly higher in patients with tumor size ≥1 cm or capsular invasion than in patients with microcarcinoma or no capsular invasion (2.3 ulU/ml *vs.* 1.4 ulU/ml, *P*=0.016 and 2.6ulU/ml *vs.* 1.7 ulU/ml, *P*=0.01). When the patients were divided into 2 groups according to TSH level (<2.5 ulU/ml and ≥2.5 ulU/ml), In the group of TSH≥2.5 ulU/ml, the percentages of patients with tumor size ≥1 cm (81%) and capsular invasion (58.2%) were significantly higher than those of patients with TSH<2.5 ulU/ml (68.3% for tumor size ≥1 and 44.0% for capsular invasion) (*P*=0.03 and *P*=0.03). The mean levels of T3 (1.4ng/ml) and T4 (81.4ng/ml) in patients with capsular invasion were lower than those of patients without capsular invasion (T3:1.6ng/ml and T4: 86.5ng/ml) (*P*=0.02). The percentages of patients with capsular invasion were significantly different among the 3 categories of T3 level (<0.9 ng/ml; ≥0.9 and <2.2 ng/ml; and ≥2.2 ng/ml, respectively) and these three groups had significant percentages of patients with capsular invasion (T3<0.9: 75%, 0.9≤T3<2.2: 45.6% and T3≥2.2: 28.6% respectively, *P*=0.003). However, other thyroid function parameters showed no significant differences in primary tumor size and capsular invasion (all *P* > 0.05). There were also no significant differences among the thyroid function parameters and affected lobes and multifocality (all *P* > 0.05).

**Table 2:** Univariate analysis on thyroid function parameters and correlative autoantibodies by primary tumor size and affected lobes of DTC

Characteristic	Primary tumor size		P	Affected lobes		P
	<1cm	≥1cm		Unilateral	Bilateral	
T3(mean±SD, ng/ml)	1.6±0.9	1.5±0.8	0.41	1.5±0.7	1.6±1.1	0.42
<0.9	7±25.0	21±75.0	0.59	19±67.9	9±32.1	0.92
≥0.9 and< 2.2	108±29.0	263±70.9		249±67.1	122±32.9	
≥2.2	8±38.1	13±61.9		15±71.4	6±28.6	
T4 (mean±SD, ng/ml)	86.9±20.9	82.9±23.5	0.11	84.1±23.2	84.2±22.5	0.95
<45	3±15.8	16±84.2	0.39	15±78.9	4±21.1	0.48
≥45 and <135	118±30.0	275±70.0		262±66.7	131±33.3	
≥135	2±25.0	6±75.0		6±75.0	2±25.0	
TSH(mean±SD, uIU/ml)	1.4±1.6	2.3±1.0	0.02	1.9±3.3	2.5±3.9	0.12
<2.5	108±31.7	233±68.3	0.03	237±69.5	104±30.5	0.07
≥2.5	15±19.0	64±81.0		46±58.2	33±41.8	
Tg(mean±SD, ng/ml)	18.8±14.1	22.3±27.9	0.19	21.9±27.9	19.9±16.1	0.43
<20	93±31.7	200±68.3	0.12	198±67.6	95±32.4	0.98
≥20	30±23.6	97±76.4		85±66.9	42±33.1	
TGAb(mean±SD, ng/ml)	1.2±1.1	1. 8±1.6	<0.05	1.6±1.4	1.7±1.7	0.63
<1	78±34.4	149±65.6	0.01	150±66.1	77±33.9	0.61
≥1	45±23.3	148±76.7		133±68.9	60±31.1	
TMAb(mean±SD, ng/ml)	1.1±1.0	1.4±1.2	0.02	1.3±1.1	1.4±1.3	0.52
<1	82±31.9	17±68.1	0.17	175±68.1	82±31.9	0.78
≥1	41 ±25.2	122±74.8		108±66.3	55±33.7	
Total	123	297		283	137	

**Table 3:** Univariate analysis of thyroid function parameters and correlative autoantibodies by capsular invasion and multifocality of DTC

Characteristic	Capsular invasion, n (%)		P	Multifocality, n (%)		P
	Yes	No		Yes	No	
T3(mean±SD, ng/ml)	1.4±0.6	1.6±0.97	0.02	1.6±0.9	1.5±0.7	0.31
<0.9	21±75.0	7±25.0	0.00	14±0.0	14±50.0	0.97
≥0.9 and< 2.2	169±45.6	202±54.4		177±47.7	194±52.3	
≥2.2	6±28.6	15±71.4		10±47.6	11±52.4	
T4 (mean±SD, ng/ml)	81.4±24.3	86.5±21.5	0.02	84.5±22.9	83.8±23.0	0.76
<45	12±63.2	7±36.8	0.32	7±36.8	12±63.2	0.51
≥45 and <135	180±45.8	213±54.2		191±48.6	202±51.4	
≥135	4±50.0	4±50.0		3±37.5	5±62.5	
TSH(mean±SD, uIU/ml)	2.6±1.1	1.7±2.9	0.01	2.4±1.2	1.8±2.7	0.11
<2.5	150±44.0	191±56.0	0.03	159±46.6	182±53.4	0.35
≥2.5	46±58.2	33±41.8		42±53.2	37±46.8	
Tg(mean±SD, ng/ml)	21.3±22.5	21.2±26.5	0.96	21.3±23.2	21.2±26.0	0.95
<20	133±45.4	160±54.6	0.49	139±47.4	154±52.6	0.87
≥20	63±49.6	64±50.4		62±48.8	65±51.2	
TGAb(mean±SD, ng/ml)	1.8±1.6	1.4±1.4	0.02	1.8±1.7	1.4±1.4	0.02
<1	91±40.1	136±59.9	0.01	97±42.7	130±57.3	0.03
≥1	105±54.4	88±45.6		104±53.9	89±46.1	
TMAb(mean±SD, ng/ml)	1.4±1.2	1.2±1.1	0.11	1.5±1.3	1.2±1.0	0.01
<1	111±43.2	146±56.8	0.09	112±43.6	145±56.4	0.035
≥1	85±52.1	78±47.9		89±54.6	74±45.4	
Total	196	224		201	219	

Our analysis demonstrated that compared with the patients with corresponding categorizes the patients with tumor size ≥1 cm (Table 2), capsular invasion and multifocality (Table 3) had higher level of TGAb (1.8 *vs.* 1.2, *P*<0.05; 1.8 *vs.* 1.4, *P* =0.02; and 1.8 vs.1.4, *P* =0.02, respectively). Additionally, the patients with tumor size ≥1 cm and multifocality had higher values of TMAb (1.4 *vs*. 1.1, *P*=0.02 and 1.5 vs. 1.2, *P*=0.01, respectively) compared with the patients with tumor size <1 cm. After the patients were subdivided into 2 categories according to their TGAb and TMAb values, the group with TGAb≥1 comprised 148 (76.7%), 105 (54.4%) and 104 (53.9%) patients with tumor size ≥1cm, capsular invasion or multifocality respectively, compared with 149 (65.6%), 91 (40.1%) and 97 (42.7%) patients with TgAb < 1 (*P*=0.01; *P*=0.005; and *P*=0.03 respectively). The group of patients with TMAb ≥ 1 more frequently presented (n=89, 54.6%) multifocality comparing the group of patients with TMAb < 1 (n =112, 43.6%).

### Comparison of preoperative thyroid function and autoantibody levels according to lymph node metastasis

Table 4 lists the associations of preoperative thyroid function and autoantibody levels with LNM. Central LNM was significantly associated with higher level of TSH (2.4 ulU/ml vs. 1.4 ulU/ml, *P* =0.001) and TGAb (1.8 vs. 1.4, *P* =0.02) in univariate analysis. Lateral LNM was significantly associated with higher level of TSH (2.7 ulU/ml vs. 1.6 ulU/ml, *P* =0.002) and TMAb (1.6 vs. 1.1, *P*<0.05). We also subdivided all the patients into 2 categories according to TSH (<2.5 ulU/ml and ≥2.5 ulU/ml), TGAb (TGAb≥1 and TGAb<1) and TMAb (TMAb≥1 and TMAb<1) and examined the differences of these parameters in LNM. In patients with TSH ≥2.5 ulU/ml, a higher prevalence of central LNM was found than those with TSH<2.5 ulU/ml (73.4% vs. 55.4%, *P* = 0.003). Moreover, there was a significant increase in the presence of lateral LNM in patients with TSH≥2.5 ulU/ml compared with TSH<2.5 ulU/ml (57.0% vs. 41.1%, *P* =0.01). In the groups TGAb≥1 and TGAb<1, the presences of central LNM (68.9% vs. 50.2%, *P*<0.05) and lateral LNM (51.8% vs. 37.4%, *P* =0.003) also significantly increased. Finally, only a significant difference in TMAb was found for lateral LNM (55.2% vs. 37.0%, *P*<0.05).

**Table 4:** Univariate analysis of thyroid function parameters and correlative autoantibodies by central and lateral LNM of DTC

Characteristic	Central LNM, n (%)		P	Lateral LNM, n (%)		P
	+	-		+	-	
T3(mean±SD, ng/ml)	1.5±0.5	1.6±0.09	0.23	1.5±0.7	1.6±0.9	0.13
<0.9	17±60.7	11±39.3	0.97	15±53.6	13±46.4	0.22
≥0.9 and< 2.2	218±58.8	153±41.2		164±44.2	207±55.8	
≥2.2	12±7.1	9±42.9		6±28.6	15±71.4	
T4 (mean±SD, ng/ml)	85.2±23.5	82.6±22.2	0.26	84.6±23.6	83.8±22.5	0.72
<45	10±52.6	9±47.4	0.74	9±47.4	10±52.6	0.18
≥45 and <135	233±59.3	160±40.7		175±44.5	218±55.5	
≥135	4±50.0	4±50.0		1±12.5	7±87.5	
TSH(mean±SD, uIU/ml)	2.5±1.4	1.4±1.4	0.00	2.7±1.8	1.6±0.8	0.00
<2.5	189±55.4	152±44.6	0.00	140±41.1	201±58.9	0.01
≥2.5	58±73.4	21±26.6		45±57.0	34±43.0	
Tg(mean±SD, ng/ml)	21.8±21.8	20.4±28.3	0.56	22.9±23.0	19.9±25.9	0.22
<20	167±57.0	126±43.0	0.25	122±41.6	171±58.4	0.13
≥20	80±63.0	47±37.0		63±49.6	64±50.4	
TGAb(mean±SD, ng/ml)	1.8±1.5	1.41±1.5	0.02	1.7±1.5	1.5±1.5	0.14
<1	114±50.2	113±49.8	<0.05	85±37.4	142±62.6	0.00
≥1	133±68.9	60±31.1		100±51.8	93±8.2	
TMAb(mean±SD, ng/ml)	1.4±1.2	1.2±1.0	0.17	1.6±1.3	1.1±1.0	<0.05
<1	143±55.6	114±44.4	0.10	95±37.0	162±63.0	<0.05
≥1	104±63.8	59±36.2		90±55.2	73±44.8	
Total	247	173		185	235	

### Multivariable analysis

Multivariable logistic regression analysis was performed to examine the preoperative thyroid function and autoantibody variables independently associated with a higher level or an increased frequency of tumor aggressive clinicopathologic features and LNM. The results of multivariable logistic regression showed that TGAb (*P*=0.01) was an independent predictive factor for primary tumor size ≥1 cm (Table 5). Furthermore, increased TSH level (*P*=0.01) and TGAb≥1 (*P* < 0.05) were associated independently with central LNM (Table 6). However, capsular invasion, multifocality and lateral LNM were not significantly associated with other preoperative thyroid function and autoantibody variables (all *P* > 0.05).

**Table 5:** Multivariable analysis on thyroid function parameters and correlative Autoantibodies by clinicopathologic characteristics

Variables	Primary tumor size		Capsular invasion		Multifocality	
	P	Exp (B) (95%CI)	P	Exp (B) (95%CI)*	P	Exp (B) (95%CI)
TSH (uIU/ml)	P=0.06	1.3 (1.0-1.7)	0.24	0.9 (0.9-1.0)		
TSH (<2.5/>2.5)	P=0.48	1.5 (0.5-4.4)	0.77	1.1 (0.6-2.2)		
TGAb	P=0.01	1.5 (1.1-2.1)	0.56	0.9 (0.8-1.2)	0.67	1.1 (0.8-1.3)
TGAb (<1/>1)	P=0.36	1.4 (0.7-2.7)	0.06	1.8 (1.0-3.2)	0.68	0.9 (0.4-1.8)
TMAb	P=0.62	0.9 (0.7-1.3)			0.37	1.2 (0.8-1.6)
TMAb (<1/>1)					0.99	1.0 (0.5-2.2)
T3 (ng/ml)			0.48	1.2 (0.8-1.8)		
T3 (<0.9/>2.2)			0.11	0.2 (0.0-1.4)		
T4 (ng/ml)			0.51	0.6 (0.2-2.5)		
T4 (45-135/>135)			0.27	1.0 (1.0-1.0)		

* adjusted by age, sex, stage, treatment, pathology, and tumor location.

**Table 6:** Multivariate logistic regression analysis thyroid function parameters and correlative autoantibodies for central and lateral LNM

Variables	Central LNM		Lateral LNM	
	P	Exp (B) (95%CI)*	P	Exp (B) (95%CI)
TSH (uIU/ml)	P=0.01	1.4 (1.1-1.8)	0.10	1.1 (1.0-1.3)
TSH (<2.5/>2.5)	P=0.38	1.6 (0.6-4.2)	0.96	1.0 (0.5-2.2)
TGAb	P=0.39	0.9 (0.7-1.1)		
TGAb (<1/>1)	P<0.05	0.4 (0.2-0.8)	0.84	0.9 (0.5-1.7)
TMAb			0.13	1.2 (0.9-1.6)
TMAb (<1/>1)			0.41	0.7 (0.4-1.5)

* adjusted by age, sex, stage, treatment, pathology, tumor size, tumor location.

## DISCUSSION

Identifying the DTC patients who need aggressive surgical treatment is a great challenge for the surgeons [11, 12]. Clinicians have been attempting to find more factors and markers to predict the prognosis of DTC patients after treatment. Thus, we evaluated four clinicopathologic features (primary tumor size, affected lobes, capsular invasion and multifocality) which significantly reflected aggressive tumor or advanced stages. Simultaneously, lymph node status was also assessed in patients with either central LNM or lateral LNM in this study. Then, we compared the preoperative thyroid function parameters (T3, T4, TSH and Tg) and TAbs (TGAb and TMAb) as they have been widely applied together with the aforementioned factors.

Serum TSH could stimulate the development of thyroid malignancy [13]. A slightly elevated preoperative TSH level was reported to be a potential predictor for the risk of thyroid carcinoma and advanced stages [14, 15], while some others still considered that the elevated TSH was not to be an independent predictor for tumor aggressiveness and poor prognosis in DTC patients [16]. In the present study, higher TSH levels were found in patients with tumor size ≥1 cm, capsular invasion, and LNM. Serum TSH level was found to be an independent risk factor for patients with central LNM. These results support the putative role of TSH in DTC development, and are in consistence with some previously published studies [17]. TSH was included in the well-characterized ATA risk stratification system as the same as using sub-analysis between TSH≥2.5 ulU/ml and TSH<2.5 ulU/ml patients. While there were no significant differences in affected lobes and multifocality, we still think that the preoperative examination of TSH has important clinical significance.

Thyroid hormones (T3 and T4) are obviously affected by TSH, and elevated TSH may usually denote to decreased thyroid function. Although some studies focused on the role of TSH, they did not take T3 and T4 levels into consideration for patients with varying thyroid function (hyperthyroidism, hypothyroidism, and euthyroidism). Recently, some researchers suggested that patients with subclinical hypothyroidism had a considerably lower rate of LNM than those with euthyroid patients (8.6% vs. 21.8%). However, further study found that T3 and T4 were not independently associated with tumor aggressiveness or prognosis in DTC in spite of reduced TSH levels and increased TSH receptor antibody values as compared with euthyroid patients [18]. Tg has been proven to be the most common tumor marker in DTC. The suitability of Tg determination for the detection of malignant thyroid remnant tissue has also improved the clinical management of DTC [19], while some false-negative Tg results have still occasionally been reported. Our results found that lower levels of T3 and T4 were only associated with capsular invasion, while no differences were found in the multivariable analysis. There were no differences between Tg and any clinicopathologic factors or LNM. According to the present findings, we might conclude that routine preoperative measurement of serum T3 and T4 is useful in determining which patients should receive operation. However, it might not helpful for predicting prognosis of DTC. The routine preoperative measurement of serum Tg is not recommended, which is the same as the ATA guideline.

Some researchers might speculate that TAb could arise independently in response to autoreceptors and release from thyrocytes damaged by cytotoxic T cells or perhaps Fas/Fas-Ligand interactions, which may cause chronic inflammation. Chronic inflammation is characterized as having a key role in the development of malignant tumors, and leads to poor prognosis [20]. On the contrary, results from multivariable analysis study suggested that intratumoral lymphocytic infiltration may be associated with better prognosis, which does not support this theory [21]. It is possible that pre-existing diffuse thyroid autoimmunity, as opposed to a specific immune response to tumor cells, could have different prognostic implications. Because of the controversial issues mentioned above, previous findings in clinical research were inconsistent. Several results have shown that TAbs was not an independent predictor of DTC prognosis [22]. Even so, most relevant studies have demonstrated that DTC may indeed lead to an autoimmune reaction characterized by circulating TAbs [23, 24]. However, whether these TAbs could be potential predictive factors of LNM in DTC has not been proved before. In agreement with many previous reports, our results demonstrated that TGAb and TMAb were higher in DTC with tumor size ≥1 cm and multifocality. The frequency of capsular invasion and central LNM was significantly related to higher TGAb, and was not associated with TMAb level.

However, the results show that lateral LNM was only affected by elevated TMAb. No significant differences were observed between the number of affected lobes and TAbs (TGAb and TMAb). Based on these results, we may conclude that TAbs may be correlated with tumor aggressiveness and prognosis in DTC, and TAbs measurement could give additional information for predicting aggressiveness and LNM. Nevertheless, it is regrettable that all these significant differences were found in univariate analysis, but not in the multivariable analysis except tumor size ≥1cm. One of the reasons could be that the renewed ATA guideline deems that it is the low-quality evidence to encourage routine preoperative measurement of TGAb. However, we still consider that TAbs may be coefficient or coexistent factors for the development of DTC, although they were not predictors for most aggressive clinicopathologic characteristics and LNM. However such findings need to be confirmed in future studies. Interestingly, when TAbs levels were divided into two groups, we found that TGAb (<1/≥1) could be an independent predictor for central LNM. While there may be some shortcomings in our study, it is worth noting that our data showed an association between TAbs and LNM in patients and DTC. Therefore, we suggest that surgeons should pay attention to the levels of TAbs, which may have potential predictive value for DTC stages.

Our study inevitably has potential limitations, which include: 1) this is a single-center study with a relatively small sample size; 2) we could not elucidate the association with serological parameters and aggressive tumor features or LNM sufficiently, such as TSH≥2.5 uIU/ml with primary tumor size <1 cm due to a very low number of cases (n=15) in our current study; and 3) It is also possible that unmeasured confounding variables and potential selection bias may have influenced present results. In conclusion, TSH level and TGAb≥1 were significantly independent predictors for central LNM, and might help make the decision of central neck dissection. However, the results from this study may be regarded as reference which can help clinicians make appropriate decisions for DTC patients.

## MATERIALS AND METHODS

### Study population

A cohort of 420 DTC patients who underwent initial surgery at Sichuan Cancer Hospital from July 2010 to July 2015 were enrolled into this study. A total of 393 papillary cases and 27 follicular cases were included. Approval from the ethics committee of the Sichuan Cancer Hospital Institutional Ethics Committee was obtained. Patients with a history of prior treatment for head and neck cancer or other thyroid malignancies were excluded from this study. Patients with highly aggressive subtypes, distant metastasis, lack of preoperative imaging studies and incomplete data were also excluded.

### Treatment protocol

All patients underwent total, subtotal or limited thyroidectomy (lobectomy with isthmusectomy) and selective central and lateral neck dissection. The central compartment (level VI) includes the pretracheal, paratracheal, and prelaryngeal lymph nodes. Elective lateral neck dissection was performed if suspicious lymph nodes were detected by imaging or clinical examinations. The thymus, the parathyroid and the recurrent laryngeal nerves were identified and preserved. Parathyroid glands which could not be preserved in situ were auto-transplanted into the ipsilateral sternocleidomastoid muscle.

### Thyroid function assay

Blood tests were obtained 1-3 days before surgery between 6:30 and 9:00 AM. Serum T4, T3, TSH and Tg were measured by a chemiluminescence immunoassay. The normal ranges for thyroid function were defined by the laboratory of the Sichuan Cancer Hospital (TSH: 0.3–5.0 uIU/mL; T3: 0.9-2.2 ng/mL; T4: 45-135 ng/mL; Tg: 0-20ng/mL). The serum TSH level was categorized into 2 levels: < 2.5 mIU/L and ≥2.5mIU/L. Because more than 95% of normal individuals have TSH levels below 2.5mIU/L, TSH value greater than 2.5mIU/L was defined as high TSH level. Tg stage was classified as < 20ng/mL and ≥20ng/mL. We also divided the patients into 3 groups at the cut-off values of 0.9ng/mL and 2.2ng/mL for the T3 level (<0.9 ng/mL, 0.9-2.2ng/mL, and > 2.2ng/mL, respectively) as well as 45ng/mL and 135ng/mL for the T4 level (<45 ng/mL, 45-135ng/mL, and > 135ng/mL, respectively).

### Measurement of thyroid autoantibodies

Preoperative levels of TgAb and TMAb were measured by a chemiluminescence immunoassay using the automated immunochemiluminescent assay. Reference ranges of variation were established by the laboratory of the Sichuan Cancer Hospital (TgAb: 0-2.1; TMAb: 0-2.1). Serum TgAb and TMAb exceeding a level of 1 were considered high serum autoantibody values. Thus, TgAb and TMAb status were further recorded as either <1 (low level) or ≥1 (high level).

### Prognostic variables of DTC

The following clinicopathologic features were analyzed for prognostic factors including aggressive tumor stage, maximal tumor size (<1 cm and ≥1 cm), extrathyroidal invasion, multifocal (multifocal lesions were defined as having two or more tumors within the thyroid) and unilateral or bilateral lesions (lesions in both lobes). Associations between central and lateral LNM and these prognostic factors were also analyzed. For quality control, analysis was performed on a randomly selected subset of 5% of the samples for all above assays. The results of the re-run samples achieved a more than 95% concordance with the original results for all assays.

### Statistical analysis

SPSS statistical package (version 17.0; Chicago, IL) was used for all statistical analysis. Risk factors according to thyroid function status and thyroid autoantibodies were evaluated using both univariate and multivariable analysis. Continuous data was represented as mean ± SD. Continuous variables were compared using the independent two-sample t-test. Chi-square test or Fisher's exact test was done to analyze categorical data as appropriate. The significant risk factors related to prognosis and LNM in univariate analysis were further included in a multivariable logistic regression analysis. Results with *P*<0.05 were regarded as having a statistical significance.

## Abbreviations

DTC: differentiated thyroid cancer
TSH: thyroid-stimulating hormone
Tab: thyroid autoantibodies
TgAb: thyroglobulin antibody
TMAb: thyroid microsomal antibody
AIT: autoimmune thyroid disease
T3 and T4: thyroid hormones
Tg: thyroglobulin
LNM: lymph node metastasis
LND: lymph node dissection.
